# Psychosocial risk for substance use in 80,000 Mexican undergraduates: mental health and academic strain

**DOI:** 10.3389/fpubh.2026.1745259

**Published:** 2026-03-19

**Authors:** Maria Fernanda Martinez-Gonzalez, Luis Adrian Alvarez-Lozada, Adrian Manuel Verdines-Pérez, Alejandro Quiroga-Garza, Guillermo Jacobo-Baca, Rodrigo Elizondo-Omaña, Santos Guzman-Lopez, Sandra Elizabeth del Río-Muñoz, Gerardo Enrique Muñoz-Maldonado, Gerardo Tamez-Gonzalez, Jaime Arturo Castillo-Elizondo, Alfonso Salinas-Zertuche

**Affiliations:** 1 Centro Universitario de Salud, Universidad Autónoma de Nuevo León, Monterrey, Mexico; 2 Department of Human Anatomy, Faculty of Medicine, Universidad Autónoma de Nuevo León, Monterrey, Mexico; 3 Universidad Autónoma de Nuevo León, Monterrey, Mexico

**Keywords:** academic stress, mental health, Mexico, POSIT, psychosocial risk factors, substance use, university students

## Abstract

**Introduction:**

In Mexico, evidence on psychosocial risk for substance use among university students is often derived from small or faculty-specific samples, limiting institutional-level comparisons across academic disciplines and sex. University-wide estimates using standardized multi-domain screening tools remain scarce.

**Methods:**

We conducted a university-wide cross-sectional study of 80,077 undergraduate students from the Autonomous University of Nuevo León (Northern Mexico). Participants completed the validated Spanish POSIT instrument, which assesses seven psychosocial domains. Psychosocial risk was operationalized as exceeding the POSIT overall risk cut-off (and domain-specific cut-offs where applicable). Group comparisons of continuous POSIT scores were performed using *t* tests and one-way ANOVA; multivariable logistic regression models were fitted to estimate adjusted odds ratios (OR) by sex and academic discipline. Construct validity was examined via confirmatory factor analysis (CFA).

**Results:**

Mean age was 21.0 years (SD = 2.9), and 51% were women. The mean POSIT total score was 13.0 (SD = 9.0), and 15.2% of students exceeded the overall risk cut-off. Domain-level risk was highest for Educative Level (61.2%) and Mental Health (49.7%), followed by Family and Peer Relationships (~40%). Women showed higher burden in Mental Health and Educative Level, while men showed higher risk in Substance Use/Abuse and Aggressive Behavior. Risk profiles differed across academic disciplines: Arts and Humanities students showed the highest overall risk (e.g., Mental Health OR ≈ 2.16 vs. other disciplines), whereas Health Sciences students showed the lowest risk across several domains.

**Conclusion:**

In this university-wide sample of 80,077 Mexican undergraduates, psychosocial risk for substance use clustered primarily in academic strain and mental health domains and varied systematically by sex and academic discipline. Findings support discipline-tailored and gender-responsive prevention strategies integrating academic support with mental health and substance-use screening and referral pathways.

## 1 Introduction

Substance use among university students has become a critical public health priority, as psychoactive substance consumption carries severe long-term consequences for personal, academic, and professional development (1). Globally, recent estimates indicate that approximately 284 million people aged 15–64 used drugs in 2020—a 26% increase over the past decade—with the highest prevalence concentrated among young adults aged 17–24 (2). These substance-related issues are major contributors to preventable morbidity and impaired functioning among young people in the Americas, particularly in low- and middle-income settings (1, 2). University students represent a uniquely vulnerable group, as the transition into higher education coincides with increased autonomy, exposure to new social environments, and academic demands that jointly shape risk trajectories (2, 3).

In Mexico, national surveillance shows substantial alcohol and drug use in adolescents and young people. The National Survey on Drug, Alcohol and Tobacco Use (ENCODAT 2016–2017) reported high levels of alcohol involvement at the population level, while the National Survey on Drug Use in Students (ENCODE 2014) documented early exposure patterns within educational settings (4, 5). Regional evidence similarly highlights that sex/gender norms and social expectations interact with risk behaviors and access to care, reinforcing inequities across the Americas (3). Beyond prevalence, a key challenge is that substance-related risk rarely emerges in isolation; it is embedded in broader psychosocial contexts such as family functioning, peer dynamics, and school engagement (6, 7).

From a theoretical perspective, academic strain can operate as a chronic stressor that influences emotional regulation and coping resources. In the transactional model of stress and coping, risk increases when perceived demands exceed available coping capacities, particularly under sustained pressures (8). In parallel, the self-medication framework proposes that some individuals may resort to substance use as an attempt to manage distressing affective states or psychological discomfort, linking mental health symptoms and consumption risk through maladaptive coping (9). These mechanisms are especially relevant in university settings, where workload, performance expectations, and adaptation to new routines may intensify stress and vulnerability.

To capture this complexity, the Problem-Oriented Screening Instrument for Teenagers (POSIT) provides a multidomain screening approach that assesses psychosocial risk in several areas related to substance use and related problems (10). Importantly, this study used the validated Spanish version developed in Mexico (UNAM), while preserving the conceptual basis of the original instrument (11). This framework allows a discipline-resolved profile of psychosocial vulnerability rather than focusing on a single outcome, supporting prevention strategies that better match the student context.

Accordingly, we aimed to map psychosocial risk patterns for substance use and related domains—particularly mental health and academic strain—across academic disciplines and by sex. We hypothesized that psychosocial risk would differ by sex and field of study, with meaningful variation in mental health and school-related domains that can inform targeted campus interventions (2–5).

## 2 Methods

### 2.1 Study design

We conducted a cross-sectional, observational, descriptive study to identify psychosocial risk factors associated with substance use and related behaviors among undergraduate students at the Autonomous University of Nuevo León (UANL), Northern Mexico.

### 2.2 Population and participants

The target population comprised all formally enrolled undergraduate students who were present during data collection and provided informed consent. For participants younger than 18 years, parental/guardian consent and student assent were obtained. Exclusion criteria were cognitive or medical impairments that could interfere with understanding or accurately answering the survey.

### 2.3 Sample size

Based on prior national evidence documenting substance-use patterns in student populations in Mexico, we assumed a 30% prevalence of substance-use risk factors to estimate the minimum required sample size with a 99% confidence level and 2% margin of error (5). The minimum required sample was 3,484 students; to accommodate potential data loss, the target sample was increased to 4,099.

### 2.4 Instrument

Psychosocial risk was assessed using the Spanish-adapted Problem-Oriented Screening Instrument for Teenagers (POSIT). The POSIT was originally developed under the U.S. National Institute on Drug Abuse adolescent assessment framework (10), and we used the Spanish validated version for Mexico developed at the Universidad Nacional Autónoma de México (UNAM) (11). The instrument evaluates seven psychosocial domains (Substance Use/Abuse, Mental Health, Family Relationships, Peer Relationships, Educative Level, Work Interest, and Aggressive Behavior) through 81 dichotomous (yes/no) items, with higher scores indicating greater psychosocial risk. Internal consistency was evaluated using Cronbach’s alpha.

To ensure the construct validity of the POSIT structure in this undergraduate sample, a confirmatory factor analysis (CFA) was conducted using maximum likelihood estimation in IBM SPSS AMOS. The model yielded a Chi-square (CMIN) of 439,243.890 (df = 3,138; *p* < 0.001) and a normed Chi-square (CMIN/DF) of 139.976. Incremental fit indices indicated modest incremental fit (CFI = 0.710; TLI = 0.700), suggesting that the seven-domain model does not reproduce the observed covariation perfectly. At the same time, absolute fit indices suggested an acceptable approximation (RMSEA = 0.042; SRMR = 0.0656). Given the very large sample size (*N* = 80,077) and the well-known sensitivity of *χ*^2^-based and incremental indices to large *N*, we prioritized absolute indices (RMSEA ≤ 0.05; SRMR ≤ 0.08) as recommended to evaluate approximate fit in large samples. Overall, the CFA supports the structural suitability of the POSIT domains for screening purposes in this population, while acknowledging some residual misfit (12, 13).

### 2.5 Data collection

Data were collected via supervised, self-administered, paper-based questionnaires in classroom settings. Standardized instructions emphasized confidentiality and the importance of honest responses. Sociodemographic variables included age, sex, and academic discipline. Responses were anonymized using numeric codes.

### 2.6 Statistical analysis

Analyses were conducted using SPSS, version 26 (IBM Corp), IBM SPSS AMOS, and R, version 4.4.3 (R Foundation for Statistical Computing). Data were screened for missingness, inconsistencies, and coding errors. Records with >10% missingness in key variables were excluded (2.64%, *n* = 2,148). No imputation was performed.

Descriptive statistics summarized sociodemographic and outcome variables. Continuous variables were described with means and standard deviations; categorical variables with frequencies and percentages. Between-group comparisons used Student’s *t* test or one-way ANOVA, as appropriate.

Binary and multivariable logistic regression models were used to identify predictors of psychosocial risk, reporting odds ratios (OR) and 95% confidence intervals (CI). Model discrimination and calibration were assessed using the area under the receiver operating characteristic curve (AUC) and the Hosmer–Lemeshow test, respectively. Bonferroni correction was applied for multiple comparisons. Predictive validity was examined using 10-fold cross-validation (*k* = 10).

### 2.7 Ethical considerations

The study was approved by the Research Ethics Committee of University Hospital “Dr. José Eleuterio González” (Approval No. AH23-00011). All procedures followed the principles of the General Health Law Regulations for Health Research in Mexico (14). Written informed consent was obtained from all participants; for minors, parental/guardian consent and participant assent were secured.

## 3 Results

### 3.1 Sample characteristics

A total of 81,225 undergraduate students participated. After excluding records with >10% missingness (2.64%, *n* = 2,148), the final analytic sample included 80,077 students, representing approximately 40% of the university population. The mean age was 21.0 years (SD = 2.9); 51% were women and 49% men. The mean score on the POSIT general scale was 13.0 (SD = 9.0), and 15.2% of participants exceeded the cut-off for psychosocial risk. Scale reliability was high: internal consistency for the overall POSIT was excellent (Cronbach’s *α* = 0.90). Descriptive characteristics and overall POSIT metrics are summarized in Table 1.

**Table 1:** Sociodemographic characteristics, mean POSIT scores, and prevalence of psychosocial risk across academic disciplines.

Variable	General	Arts and Humanities	Agronomic and Veterinary Sciences	Health Sciences	Natural Sciences	Social Sciences	Engineering and Technology
*N* (%)	80,077 (100)	4,684 (5.8)	1,458 (1.8)	9,645 (12)	6,545 (8.2)	31,784 (39.7)	25,961 (32.4)
Age	21.02 ± 2.85	21.12 ± 3.01	21.15 ± 3	21.04 ± 2.82	21.09 ± 2.59	20.83 ± 3	21.21 ± 2.69
Sex							
Women	40,777 (51)	3,333 (71.2)	910 (62.4)	6,763 (70.1)	2,729 (41.7)	18,290 (57.5)	8,752 (33.7)
Men	39,300 (49)	1,351 (28.8)	548 (37.6)	2,882 (29.9)	3,816 (58.3)	13,494 (42.5)	17,209 (66.3)
POSIT score	13.5 ± 9.22	15.79 ± 9.8	14.78 ± 9.7	12.85 ± 8.86	15.21 ± 9.68	13.04 ± 9.19	13.38 ± 9.04
Risk rate	12,204 (15.2)	1,062 (22.7)	287 (19.7)	1,336 (13.9)	1,303 (19.9)	4,498 (14.2)	3,718 (14.3)
POSIT subscales							
Substance use/abuse							
POSIT	0.49 ± 1.49	0.48 ± 1.46	0.54 ± 1.42	0.39 ± 1.25	0.51 ± 1.58	0.5 ± 1.52	0.52 ± 1.52
Risk	16,612 (20.7)	914 (19.5)	334 (22.9)	1,773 (18.4)	1,352 (20.7)	6,658 (20.9)	5,581 (21.5)
Mental health							
POSIT	3.59 ± 3.52	5.03 ± 3.89	4.12 ± 3.71	3.65 ± 3.48	4.27 ± 3.75	3.43 ± 3.47	3.3 ± 3.37
Risk	39,815 (49.7)	3,144 (67.1)	799 (54.8)	5,033 (52.2)	3,692 (56.4)	15,518 (48.8)	11,629 (44.8)
Family relationships							
POSIT	2.25 ± 2.24	2.48 ± 2.32	2.39 ± 2.33	2.18 ± 2.24	2.4 ± 2.24	2.21 ± 2.23	2.24 ± 2.22
Risk	32,084 (40.1)	2,030 (43.3)	637 (43.7)	3,674 (38.1)	2,789 (42.6)	12,586 (39.6)	10,368 (39.9)
Peer relationships							
POSIT	0.67 ± 1.06	0.64 ± 1.06	0.65 ± 1.03	0.59 ± 0.97	0.69 ± 1.09	0.68 ± 1.07	0.68 ± 1.06
Risk	32,206 (40.2)	1,745 (37.3)	577 (39.6)	3,619 (37.5)	2,641 (40.4)	12,978 (40.8)	10,646 (41)
Educative level							
POSIT	3.49 ± 2.94	4.29 ± 3.2	4 ± 3.19	3.45 ± 2.88	4.23 ± 3.16	3.21 ± 2.83	3.47 ± 2.91
Risk	49,015 (61.2)	3,289 (70.2)	968 (66.4)	5,776 (59.9)	4,477 (68.4)	18,810 (59.2)	15,695 (60.5)
Aggressive behavior							
POSIT	1.80 ± 1.96	2.02 ± 2.01	1.94 ± 2.01	1.62 ± 1.83	2.03 ± 2.05	1.77 ± 1.98	1.8 ± 1.93
Risk	11,930 (14.9)	806 (17.2)	243 (16.7)	1,189 (12.3)	1,172 (17.9)	4,484 (14.1)	4,036 (15.5)
Work interest							
POSIT	0.85 ± 0.75	0.86 ± 0.79	0.91 ± 0.75	0.68 ± 0.72	0.83 ± 0.79	0.89 ± 0.75	0.87 ± 0.74
Risk	2,288 (2.9)	166 (3.5)	49 (3.4)	164 (1.7)	232 (3.5)	937 (2.9)	740 (2.9)

Continuous variables are presented as mean ± standard deviation. Categorical variables are presented as *N* (%). POSIT, Problem-Oriented Screening Instrument for Teenagers.

### 3.2 Differences by academic discipline

**Figure 1:**
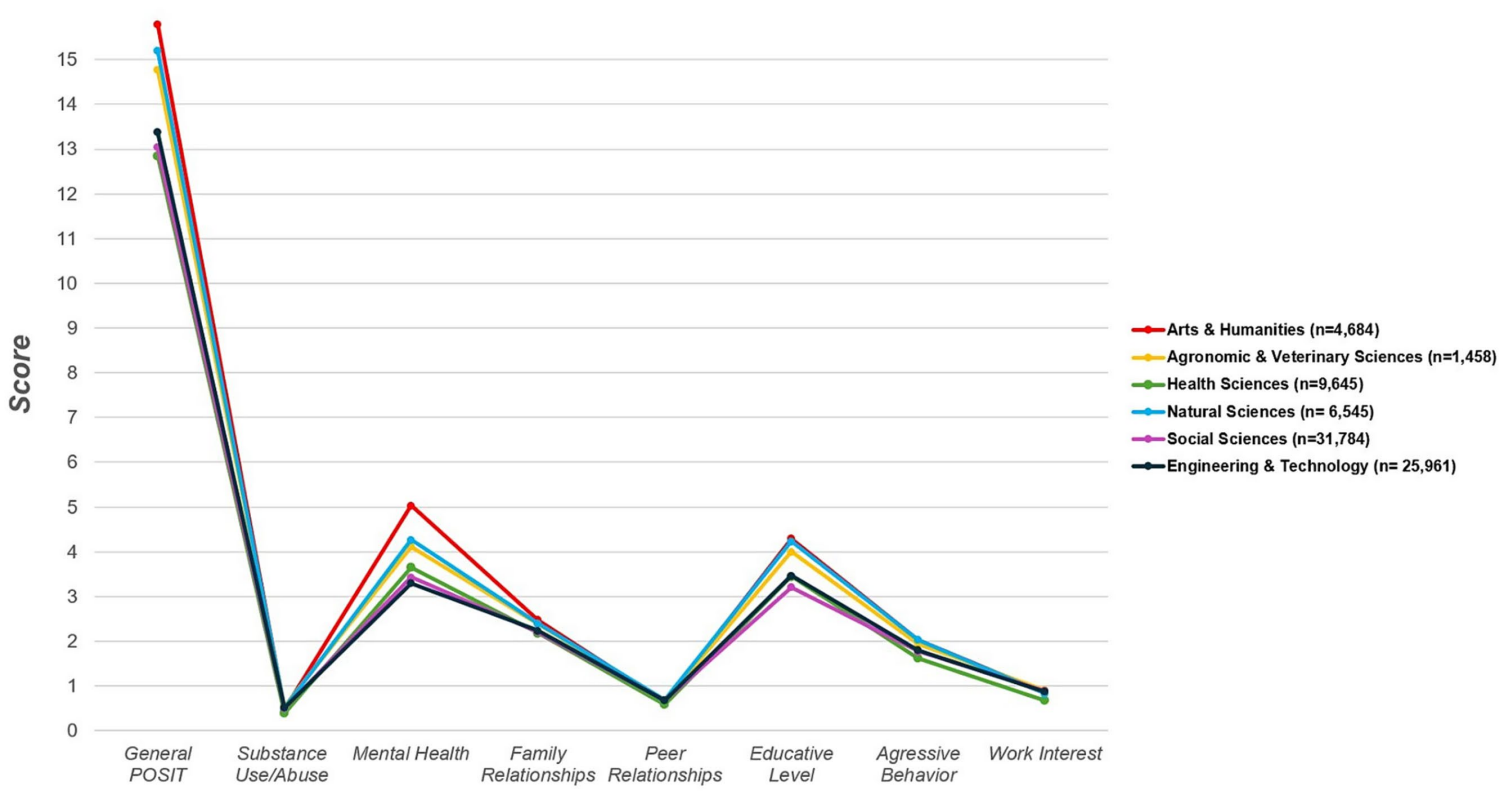
Mean POSIT scores across academic disciplines. Displays mean scores of the POSIT general scale and its subscales by field of study. Higher values indicate greater psychosocial risk. POSIT, Problem-Oriented Screening Instrument for Teenagers.

Risk profiles varied across academic disciplines. Students in Arts and Humanities exhibited the highest average POSIT score (15.8), followed by Natural Sciences (15.2) and Agronomic and Veterinary Sciences (14.8). In contrast, Health Sciences students showed the lowest mean score (12.9), consistent with a comparatively favorable psychosocial profile. These between-field differences are detailed in Table 1 and visualized by domain in the trend plot (Figure 1). The pattern was evident despite substantial within-field variability expected in a university-wide sample.

### 3.3 Domain-level prevalence

Across POSIT domains, the greatest proportion meeting risk criteria appeared in Educative Level (61.2%) and Mental Health (49.7%). Risk in Family Relationships and Peer Relationships was observed in approximately 40% of students. For Substance Use/Abuse, 20.7% exceeded the risk cut-off, whereas Aggressive Behavior affected 14.9%. The lowest prevalence was noted for Work Interest (2.9%). Overall, vulnerabilities clustered in academic strain and internalizing symptoms rather than externalizing behaviors, with domain scores by field illustrated in Figure 1.

### 3.4 Sex differences

**Figure 2:**
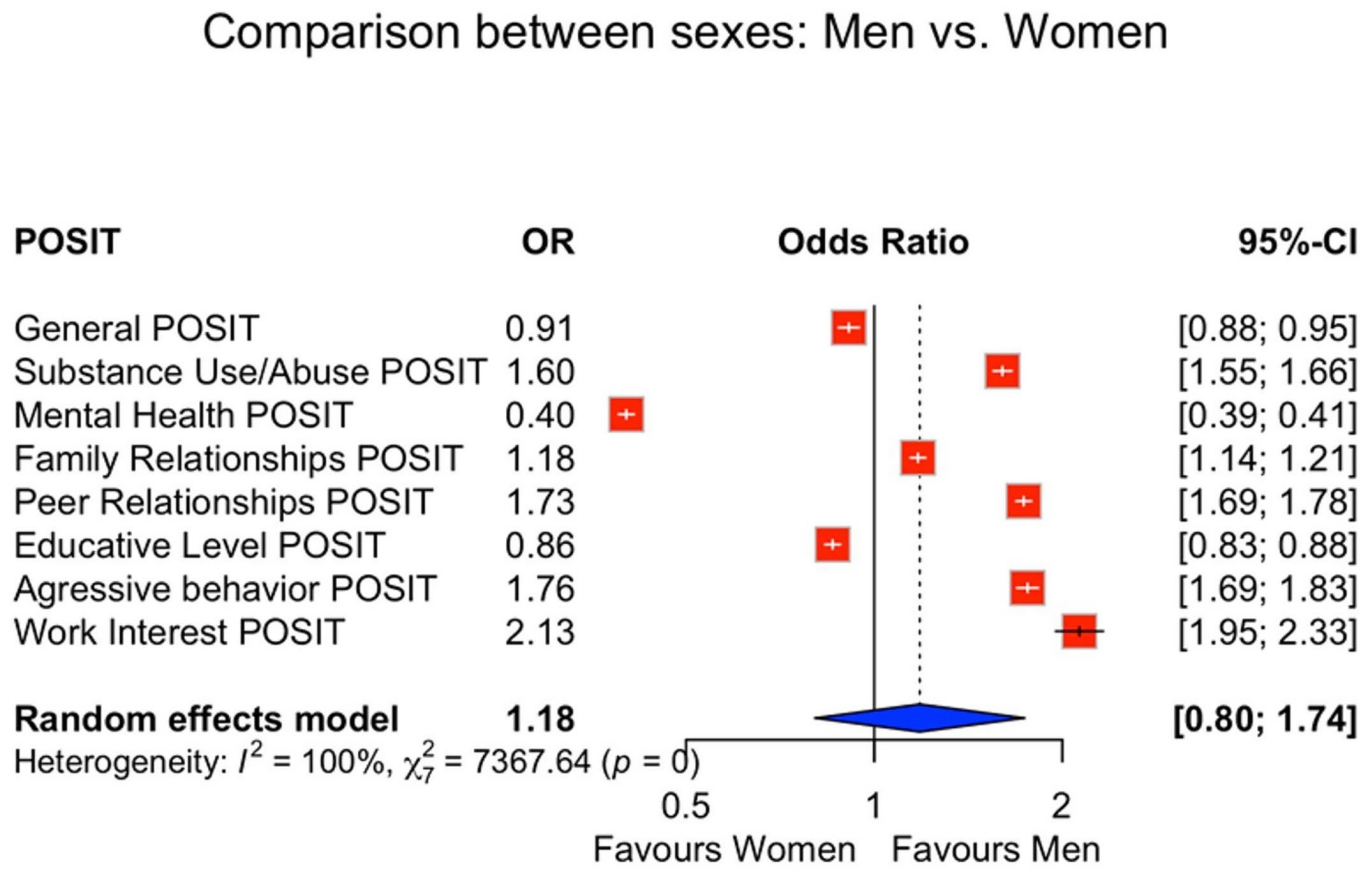
Odds ratios comparing men and women. Logistic regression models comparing risk classification in the POSIT general scale and subscales by sex. Models adjusted for age and academic discipline. OR, odds ratio; CI, confidence interval; POSIT, Problem-Oriented Screening Instrument for Teenagers.

Women scored higher in Mental Health, Family Relationships, and Educative Level, whereas men scored higher in Substance Use/Abuse, Peer Relationships, and Aggressive Behavior. For the general POSIT classification, women were less likely than men to exceed the cut-off (OR = 0.91, 95% CI 0.88–0.95). Men showed higher odds of risk in Substance Use/Abuse (OR = 1.60, 95% CI 1.55–1.66), Peer Relationships (OR = 1.73, 95% CI 1.69–1.78), Aggressive Behavior (OR = 1.76, 95% CI 1.69–1.83), and Work Interest (OR = 2.13, 95% CI 1.95–2.33). In contrast, men showed lower odds for Mental Health (OR = 0.40, 95% CI 0.39–0.41) and Educative Level (OR = 0.86, 95% CI 0.83–0.88) (Figure 2). Distributional contrasts for domain scores by sex are provided in the supplement (Supplementary Figure S1).

### 3.5 Multivariable models by academic discipline

**Figure 3:**
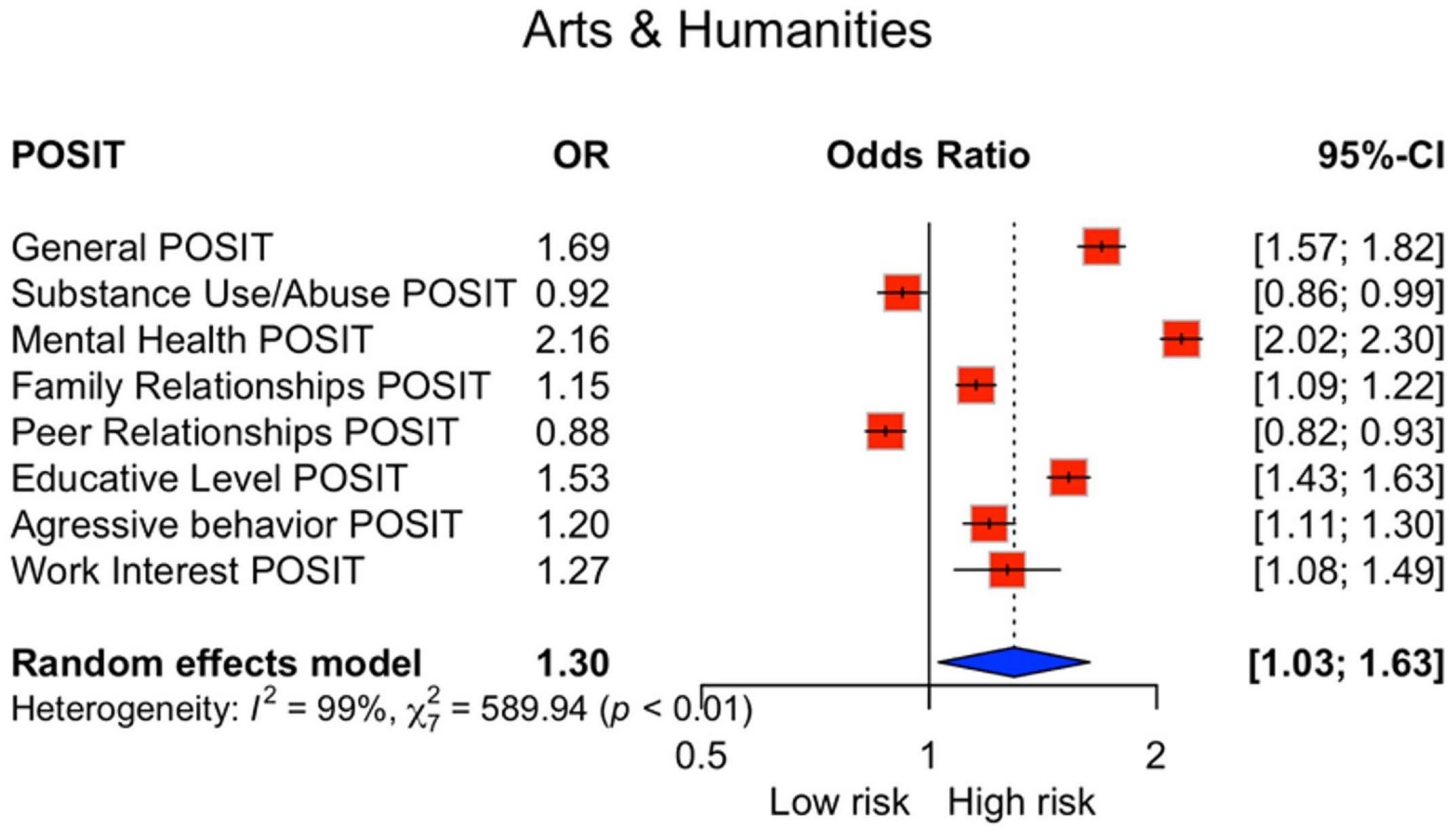
Odds ratios for students in Arts and Humanities. Odds ratios comparing Arts and Humanities students versus all other disciplines across POSIT subscales. Models adjusted for age and sex. OR, odds ratio; CI, confidence interval; POSIT, Problem-Oriented Screening Instrument for Teenagers.

**Figure 4:**
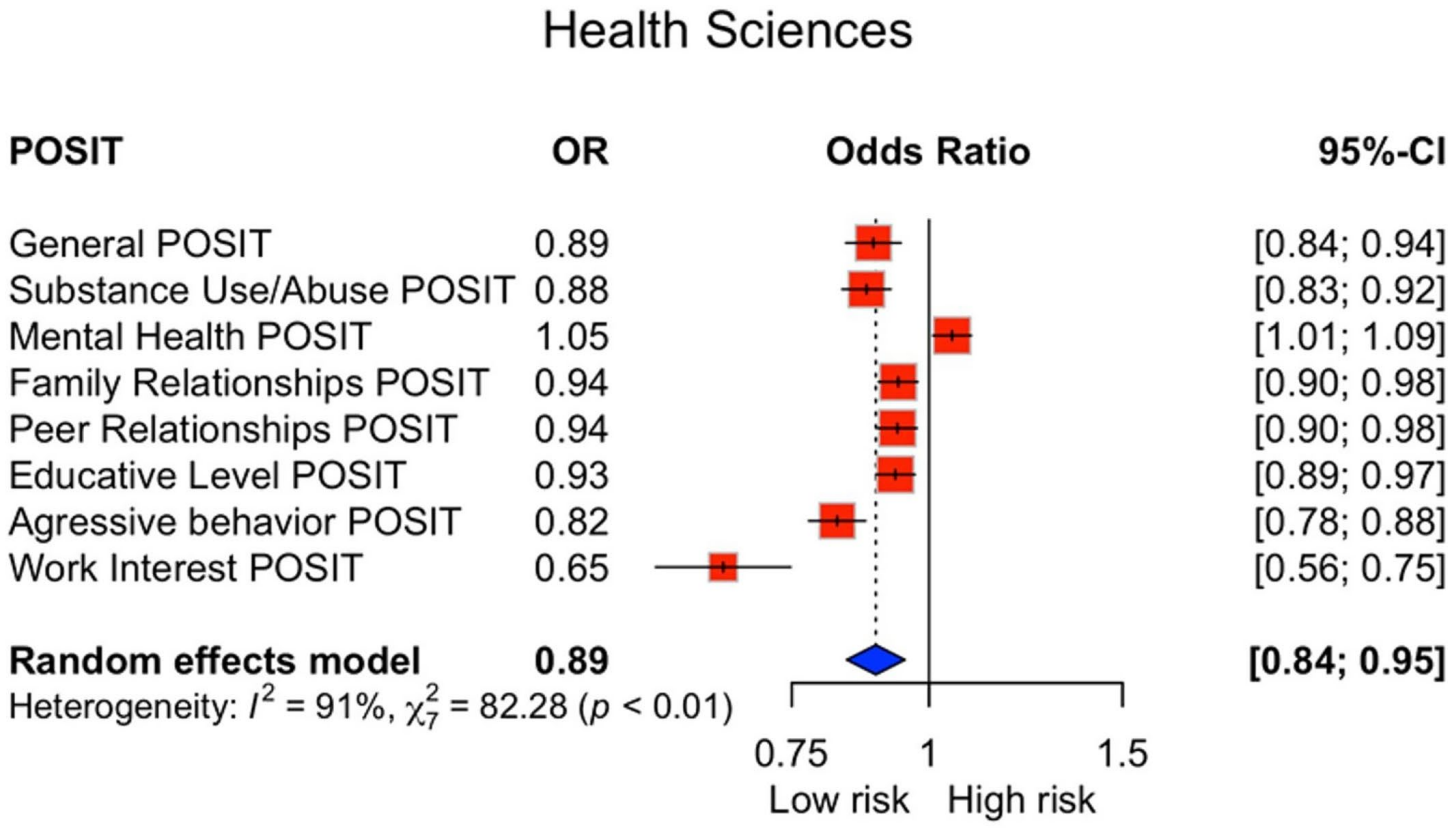
Odds ratios for students in Health Sciences. Odds ratios comparing Health Sciences students versus all other disciplines. Models adjusted for age and sex. Health sciences students showed the lowest risk profile across domains, with reduced odds in substance use/abuse (OR = 0.88) and aggressive behavior (OR = 0.82). OR, odds ratio; CI, confidence interval; POSIT, Problem-Oriented Screening Instrument for Teenagers.

Multivariable logistic regression comparing each field against all others showed that Arts and Humanities presented the highest odds of risk in Mental Health (OR = 2.16, 95% CI 2.02–2.30) and elevated odds in Educative Level (OR = 1.53, 95% CI 1.43–1.63) (Figure 3). Health Sciences displayed the lowest risk profile across most domains, including reduced odds for Substance Use/Abuse (OR = 0.88, 95% CI 0.83–0.92) and Aggressive Behavior (OR = 0.82, 95% CI 0.78–0.88) (Figure 4). Distinct patterns for Agronomic and Veterinary Sciences, Natural Sciences, Social Sciences, and Engineering and Technology are presented in the Supplementary Figures S2–S5.

### 3.6 Correlation structure

Correlation analyses showed strong positive associations between the general POSIT score and the Mental Health (*r* = 0.82), Educative Level (*r* = 0.82), and Family Relationships (*r* = 0.76) domains, all *p* < 0.001. Correlations with other domains were positive and statistically significant but lower in magnitude. These findings support the centrality of mental health and academic strain in overall psychosocial burden (Supplementary Figure S6).

### 3.7 Summary of main patterns

We observed (i) substantial overall psychosocial burden, with one in six students exceeding the general cut-off; (ii) pronounced domain-level vulnerabilities in Educative Level and Mental Health; (iii) systematic sex differences (higher internalizing burden among women and higher substance-related/externalizing burden among men); and (iv) marked heterogeneity by academic discipline, with Arts and Humanities showing comparatively elevated risk and Health Sciences consistently lower risk across several domains (Table 1) and reinforced by the figure set (Figures 1–4; Supplementary Figures S1–S6).

## 4 Discussion

### 4.1 Interpretation and novelty

This large-scale, cross-sectional survey, the largest of its kind conducted in Mexico involving over 80,000 students, establishes a substantial burden of psychosocial risk in the Northern Mexican university population. While confirming that one in six students screens at psychosocial risk, our findings advance the literature by delivering a unique, discipline-resolved risk profile across sex and academic field: the burden concentrates in Educational and Mental Health domains and varies systematically by sex and discipline (Figures 2–4; Supplementary Figures S1–S6). The unprecedented scale with domain- and field-specific estimates, together with the strong co-occurrence of educational strain and mental-health symptoms—an actionable pattern often missed by single-domain screenings—supports an integrated view linking learning environments, affective symptoms, and substance-use vulnerability within a common framework (1, 15, 16).

### 4.2 Mechanistic and theoretical interpretation

The Educational–Mental Health clustering is consistent with stress–coping and self-regulation models in emerging adulthood, wherein academic load, sleep disruption, and affective symptoms reinforce one another, potentially channeling substance use as maladaptive coping (8, 9, 15, 16). Sex differences, with higher internalizing burden among women and higher substance-related or externalizing burden among men, align with gendered socialization and masculine-norm scripts that normalize risk-taking and discourage help-seeking, which may also foster underreporting of emotional symptoms among men (3, 4, 17–20).

This between-discipline heterogeneity aligns with evidence showing higher psychological distress in expressive fields. Multi-site studies report greater burdens of mental health problems and suicidality in Arts and Humanities compared with other disciplines, and work on sleep and performance indicates that students in arts-oriented programs show more dysfunctional sleep beliefs and performance-anxiety–related symptoms than their peers (21–23). In contrast, Health Sciences programs typically include more structured curricular routines, higher health literacy, and formalized peer or mentor supports, which are associated with lower anxiety and improved coping among undergraduates (24–26). Taken together, these patterns support the hypothesis that distinct academic ecologies—more evaluative and performance-salient in creative disciplines and more structured and support-rich in Health Sciences—help shape both risk and buffering mechanisms in university populations.

Regarding the low prevalence observed in the Work Interest domain (2.9%), several factors may explain this finding. On one hand, it could represent a real phenomenon in this cohort, suggesting high vocational orientation or a lack of immediate labor-related conflicts while still in training. On the other hand, this low score may reflect a “floor effect,” where the current POSIT items—originally developed for teenagers—may lack the sensitivity or age-appropriateness required to capture the more complex professional and financial stressors faced by university students in later stages of their degrees. Future iterations of the instrument for higher education should consider refining these items to better reflect the current socioeconomic realities of young adults entering the workforce.

### 4.3 Comparison with prior evidence

Our patterning accords with international reports establishing that the early onset of substance use is consistently linked to adverse long-term outcomes, including anxiety, depression, antisocial behavior, academic underperformance, and a heightened propensity for later addiction. This risk trajectory is amplified by the inherent neurodevelopmental sensitivity during youth, a period of heightened cerebral vulnerability (1, 15, 27).

Mexican studies during and after the COVID-19 period document elevated anxiety, depression, and substance use in university students and medical trainees, with social support and physical activity showing protection (28–30). The sex gradient in substance use and externalizing outcomes replicates regional findings and is consistent with higher male suicide mortality in Mexico and the Americas, which underscores unmet needs among men (4, 17–20, 31, 32). Our results extend prior work by mapping risk by academic field at scale, confirming comparatively lower odds across several domains in Health Sciences and higher odds in Arts and Humanities, and detailing intermediate, domain-specific profiles in other fields—a level of granularity seldom reported in large Latin American cohorts (21).

### 4.4 Causal inferences and generalization

Directionality cannot be inferred from cross-sectional data. Academic strain may exacerbate mental-health symptoms, and pre-existing symptoms may impair academic performance. Substance use can act as both antecedent and consequence. Even so, the consistency of observed gradients with theory and prior evidence supports cautiously generalizable principles: internalizing–academic coupling is central, gendered norms shape risk expression and service use, and disciplinary context modifies both exposures and buffers (1, 8, 9, 15, 16, 27–34).

### 4.5 Implications for university policy and practice

Findings justify a stepped, data-driven campus health model that is multidisciplinary, gender-responsive, and discipline-tailored. First, implement brief, periodic screening for mental health, academic strain, and substance use at enrollment and key milestones, with low-threshold digital triage to counseling and academic advising (16, 28–30, 34). Second, integrate academic and mental-health supports, co-locating tutoring and study-skills services with counseling, and prioritize students with concurrent Educational and Mental Health risk as suggested by the correlation structure (Supplementary Figure S6) (15, 16). Third, tailor programming by field: in Arts and Humanities emphasize affect regulation, workload planning, and stigma reduction around help-seeking (Figure 3); in Engineering and Technology pair motivational interviewing and substance-use prevention with initiatives that normalize emotional literacy; in Health Sciences maintain protective practices such as mentoring and structured rotations and monitor burnout (Figure 4; Supplementary Figures S2–S5) (21–26).

Fourth, adopt gender-responsive strategies, including male-friendly entry points to care (for example, drop-in counseling and peer mentors), messaging that challenges norms equating risk-taking with status, and active case finding for emotional symptoms in men, while expanding timely access to evidence-based treatments and academic accommodations for women with internalizing symptoms (3, 17–20, 32). Fifth, strengthen the family–peer ecosystem: peer programs improve wellbeing but may not fully buffer family dysfunction, therefore complement them with family-communication resources and, where feasible, family-inclusive counseling (35–37). Sixth, shape the campus substance-use environment through consistent policy enforcement, scaled brief interventions, recovery-friendly options, and coordination with community services for stepped care (1, 15, 31, 32).

Seventh, establish a robust framework for ethical screening and data governance. To prevent stigmatization, screening results must be used strictly for supportive purposes, ensuring that detection is always followed by clear, predefined referral pathways to clinical or psychological care. Furthermore, the institution must maintain rigorous data protection protocols, including anonymization via numeric coding, restricted access to authorized personnel, and transparent accountability standards for the secondary use of large-scale institutional datasets.

### 4.6 Strengths and limitations; future directions

Strengths include the unprecedented sample size, broad disciplinary coverage, and use of a validated instrument that enables domain-resolved profiling and robust subgroup analyses, enhancing precision and generalizability within the university context. Limitations include the cross-sectional design, which precludes inferring causality or temporal order. Reliance on self-report may introduce recall and social-desirability biases. Furthermore, the dichotomous (yes/no) POSIT format does not capture the severity, frequency, or duration of symptoms.

Potential residual confounding remains, such as socioeconomic status, prior mental health treatment, and campus-level academic culture. Records with >10% missingness were excluded (2.64%; *n* = 2,148), and no imputation was performed. Selection bias may exist due to classroom recruitment and voluntary participation, potentially underrepresenting students with higher stress or absenteeism. Additionally, academic semester was unavailable, and disciplines were grouped into broad categories, which may mask within-field heterogeneity and requires caution when interpreting risk by field. Finally, while models showed adequate discrimination, future reports should include specific metrics such as Pseudo-R^2^ and classification matrices to further detail model performance.

Future studies should use longitudinal designs, incorporate finer academic granularity and complementary dimensional measures, and evaluate stepped-care interventions stratified by sex and discipline, including academic outcomes such as retention and time-to-degree. These priorities are reinforced by recent evidence documenting persistent emotional distress among Mexican university students and by regional data showing a substantial ongoing mental health burden across the Americas (40, 41).

### 4.7 Translational significance

By providing a discipline-resolved risk map for more than 80,000 students, this study offers universities actionable baselines to allocate resources, tailor interventions, and monitor progress toward equitable wellbeing and performance. Given documented treatment gaps and mortality patterns in the region, translating these findings into structured and evaluated campus strategies is both feasible and urgent (31, 32, 38, 39).

## 5 Conclusion

This study delivers a discipline-resolved, population-scale map of psychosocial risk in Mexican higher education, clarifying how educational strain and internalizing symptoms align across sex and academic fields. Rather than emphasizing prevalence alone, the contribution is a framework that locates risk where universities can intervene: at the intersection of learning conditions, mental health, and substance-use vulnerability.

For practice, the evidence supports a campus strategy that is data-driven and stepped: brief universal screening with rapid referral; co-located academic and mental-health services; gender-responsive outreach that normalizes help-seeking among men; and discipline-tailored programs that reflect distinct profiles in Arts and Humanities, Engineering/Technology, and Health Sciences. Strengthening peer mentoring, addressing the campus substance-use environment, and using dashboards to track outcomes can operationalize this approach.

## Data Availability

The raw data supporting the conclusions of this article are available from the corresponding author upon reasonable request, provided that such access complies with the applicable privacy and data protection requirements for minor and student participants.

## Supplementary material

SUPPLEMENTARY FIGURE S1 Mean POSIT scores by sex. Comparison of men and women across the POSIT general scale and subscales. Higher scores indicate greater psychosocial risk. POSIT, Problem-Oriented Screening Instrument for Teenagers.

SUPPLEMENTARY FIGURE S2 Odds ratios for students in Agronomic and Veterinary Sciences. Odds ratios comparing Agronomic and Veterinary Sciences students versus all other disciplines. Models adjusted for age and sex. OR, odds ratio; CI, confidence interval; POSIT, Problem-Oriented Screening Instrument for Teenagers.

SUPPLEMENTARY FIGURE S3 Odds ratios for students in Natural Sciences. Odds ratios comparing Natural Sciences students versus all other disciplines. Models adjusted for age and sex. OR, odds ratio; CI, confidence interval; POSIT, Problem-Oriented Screening Instrument for Teenagers.

SUPPLEMENTARY FIGURE S4 Odds ratios for students in Social Sciences. Odds ratios comparing Social Sciences students versus all other disciplines. Models adjusted for age and sex. OR, odds ratio; CI, confidence interval; POSIT, Problem-Oriented Screening Instrument for Teenagers.

SUPPLEMENTARY FIGURE S5 Odds ratios for students in Engineering and Technology. Odds ratios comparing Engineering and Technology students versus all other disciplines. Models adjusted for age and sex. OR, odds ratio; CI, confidence interval; POSIT, Problem-Oriented Screening Instrument for Teenagers.

SUPPLEMENTARY FIGURE S6 Correlation matrix between POSIT domains. Correlation matrix showing Pearson correlation coefficients between the POSIT general score and subscales. All associations were statistically significant (*p* < 0.001). POSIT, Problem-Oriented Screening Instrument for Teenagers; OR, odds ratio; CI, confidence interval.
