# Supplementary figures and images for: Psychosocial risk for substance use in 80,000 Mexican undergraduates: mental health and academic strain
Source: Front Public Health. 2026 Mar 19;14:1745259. doi: 10.3389/fpubh.2026.1745259 (PMC13043337; doi:10.3389/fpubh.2026.1745259)

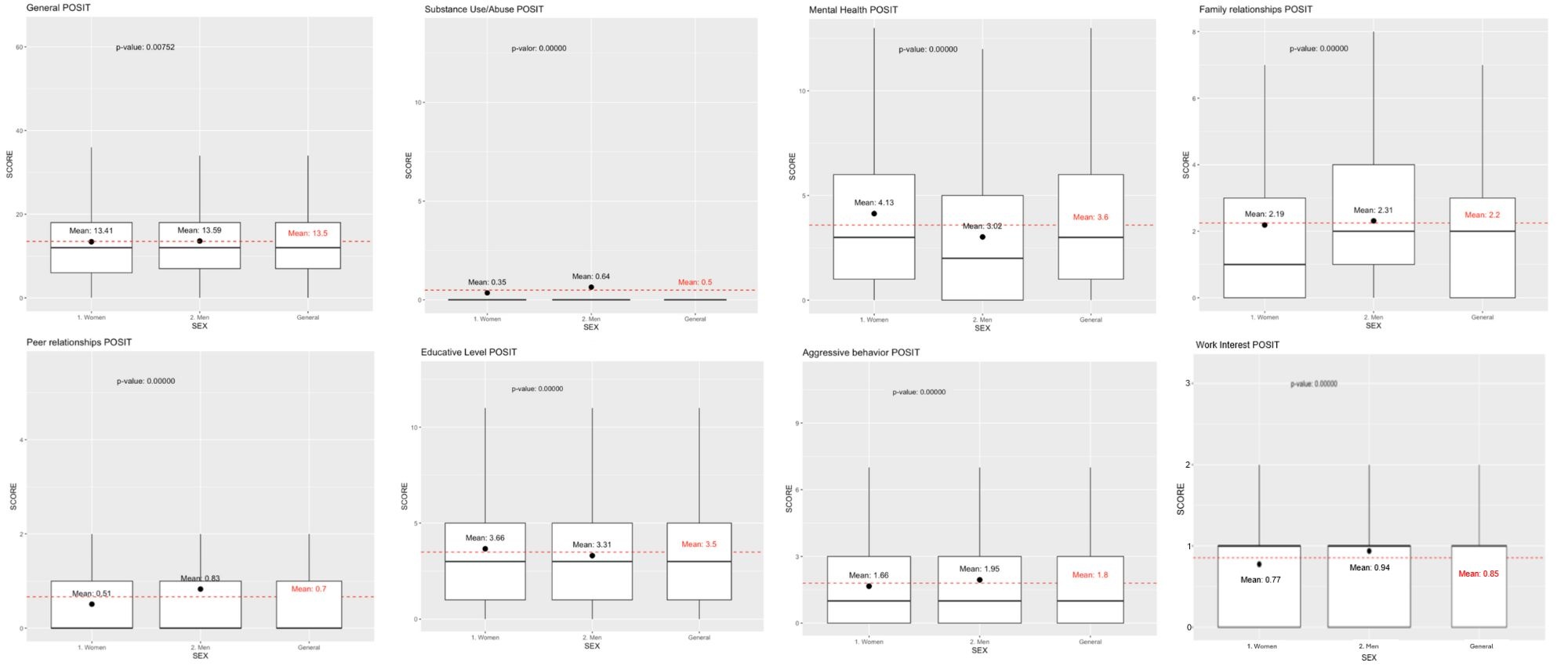

Supplement: SUPPLEMENTARY FIGURE S1 — Mean POSIT scores by sex. Comparison of men and women across the POSIT general scale and subscales. Higher scores indicate greater psychosocial risk. POSIT, Problem-Oriented Screening Instrument for Teenagers. [file Image_1.JPEG]

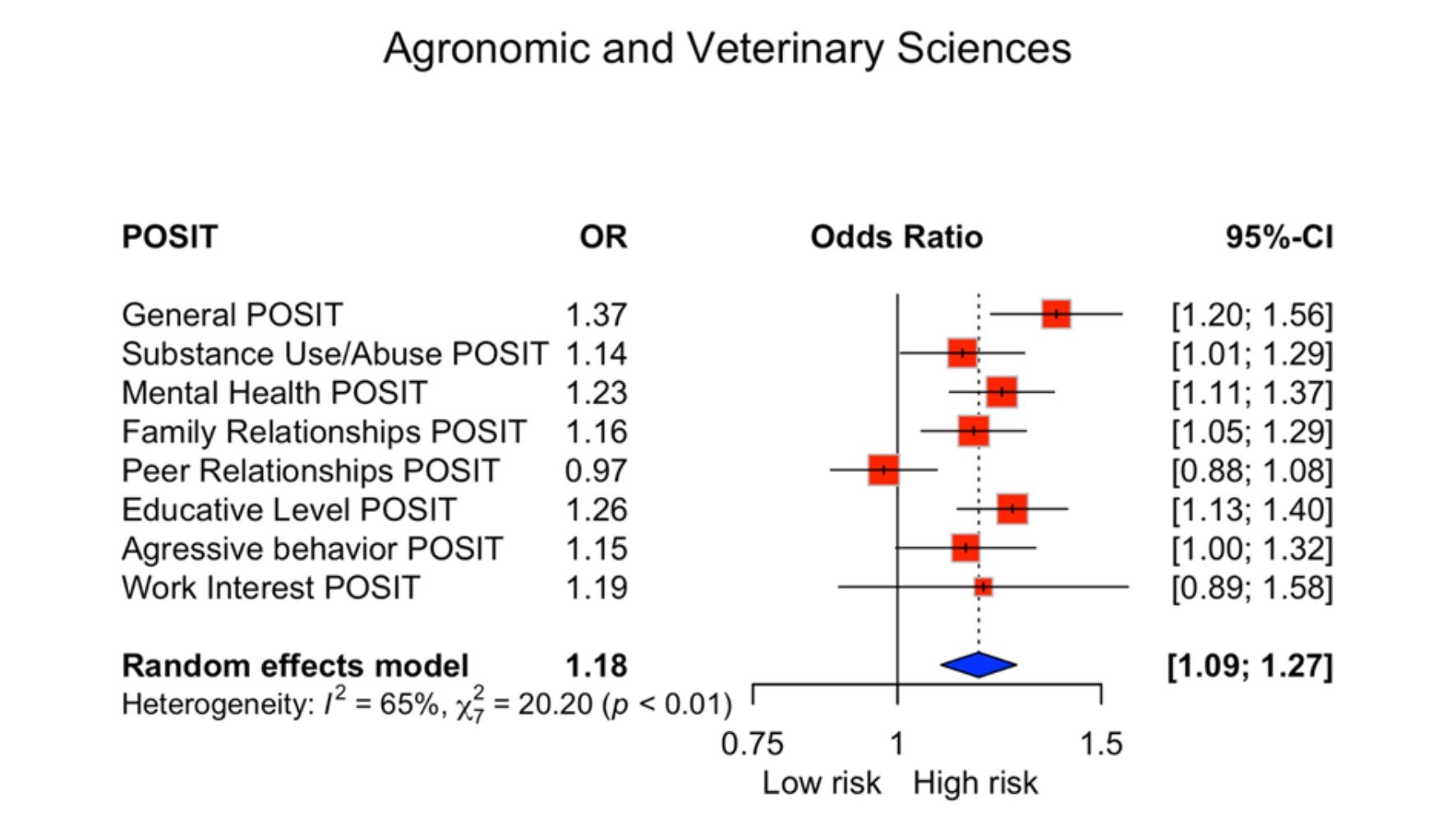

Supplement: SUPPLEMENTARY FIGURE S2 — Odds ratios for students in Agronomic and Veterinary Sciences. Odds ratios comparing Agronomic and Veterinary Sciences students versus all other disciplines. Models adjusted for age and sex. OR, odds ratio; CI, confidence interval; POSIT, Problem-Oriented Screening Instrument for Teenagers. [file Image_2.JPEG]

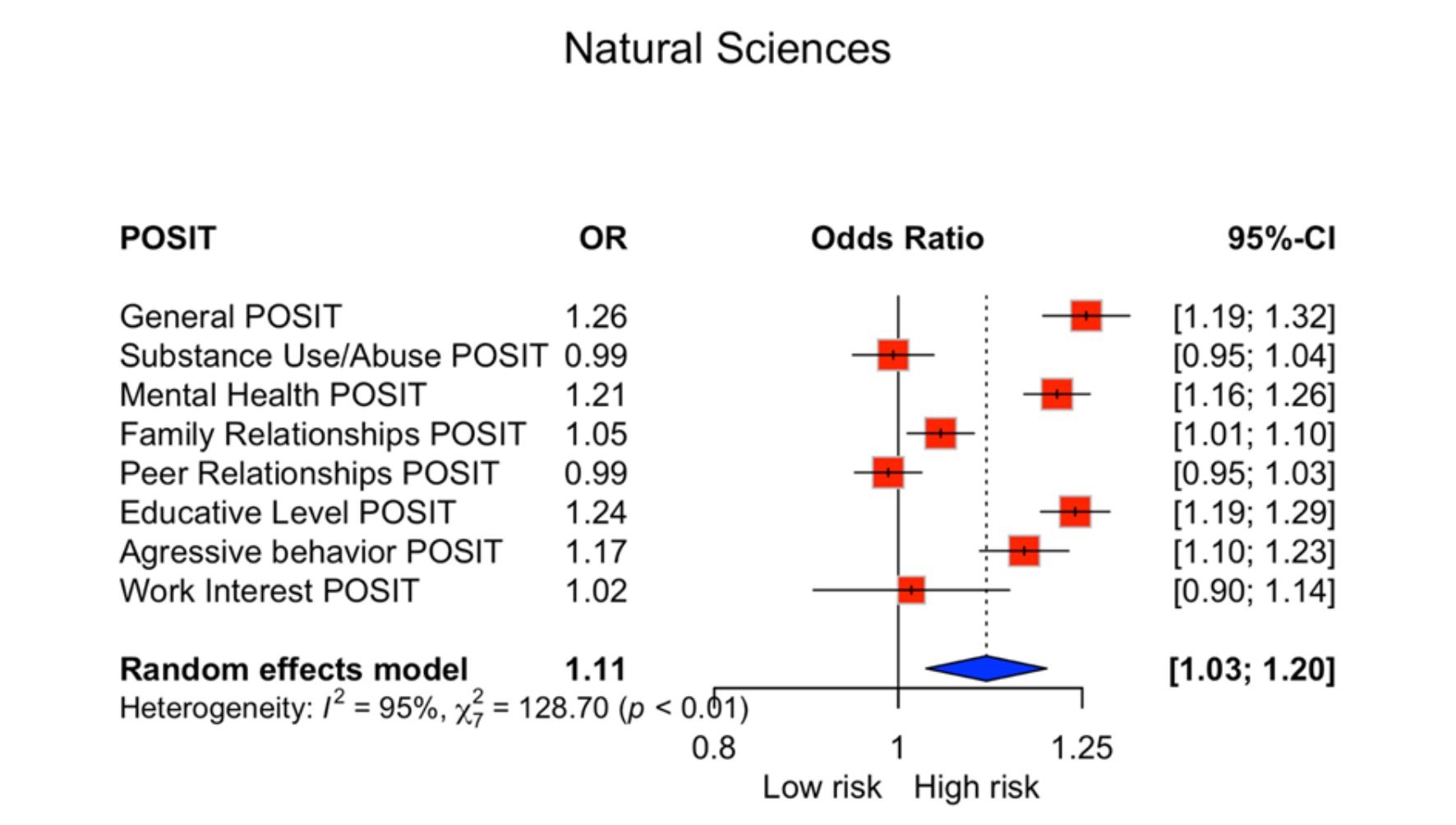

Supplement: SUPPLEMENTARY FIGURE S3 — Odds ratios for students in Natural Sciences. Odds ratios comparing Natural Sciences students versus all other disciplines. Models adjusted for age and sex. OR, odds ratio; CI, confidence interval; POSIT, Problem-Oriented Screening Instrument for Teenagers. [file Image_3.JPEG]

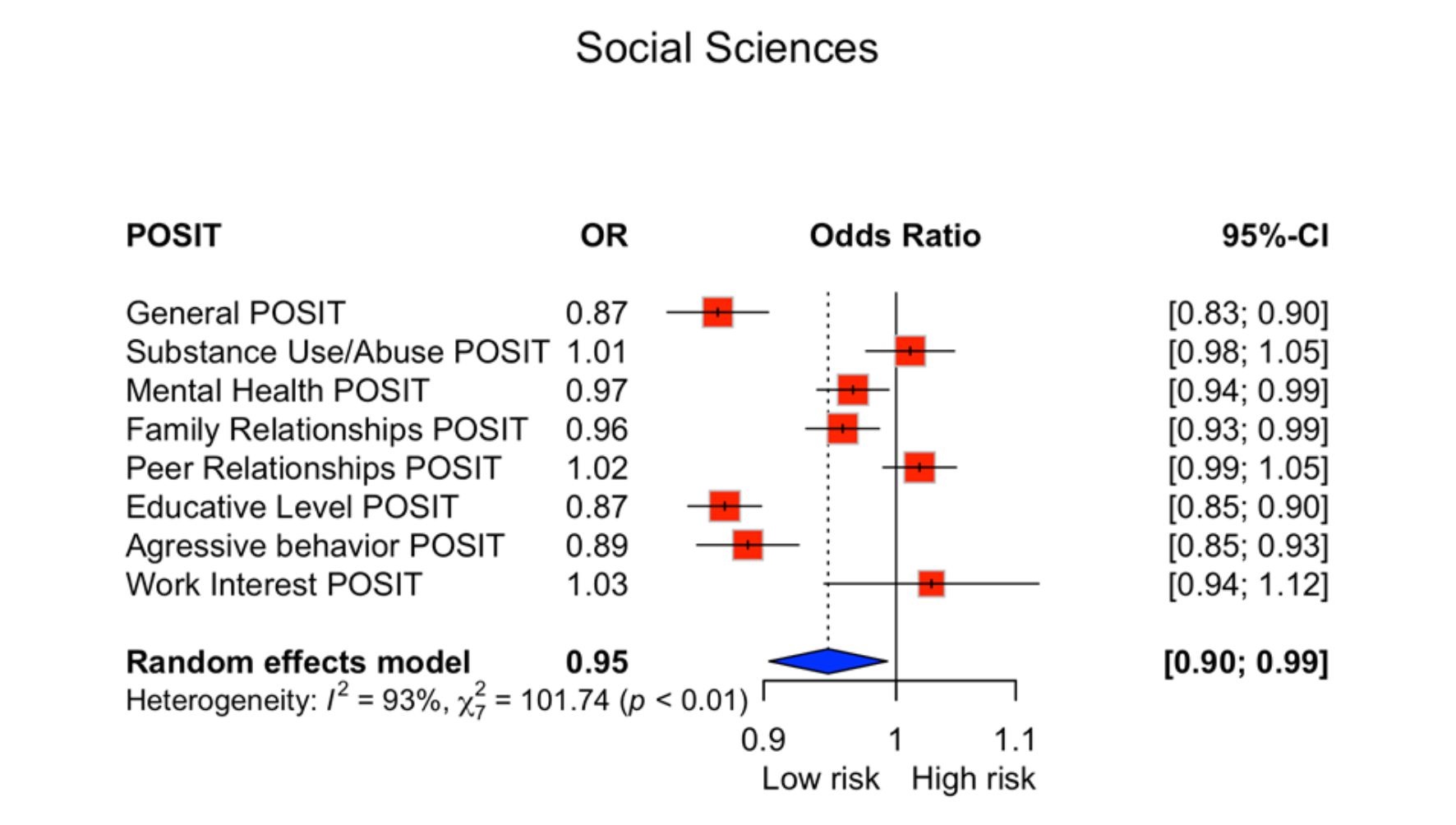

Supplement: SUPPLEMENTARY FIGURE S4 — Odds ratios for students in Social Sciences. Odds ratios comparing Social Sciences students versus all other disciplines. Models adjusted for age and sex. OR, odds ratio; CI, confidence interval; POSIT, Problem-Oriented Screening Instrument for Teenagers. [file Image_4.JPEG]

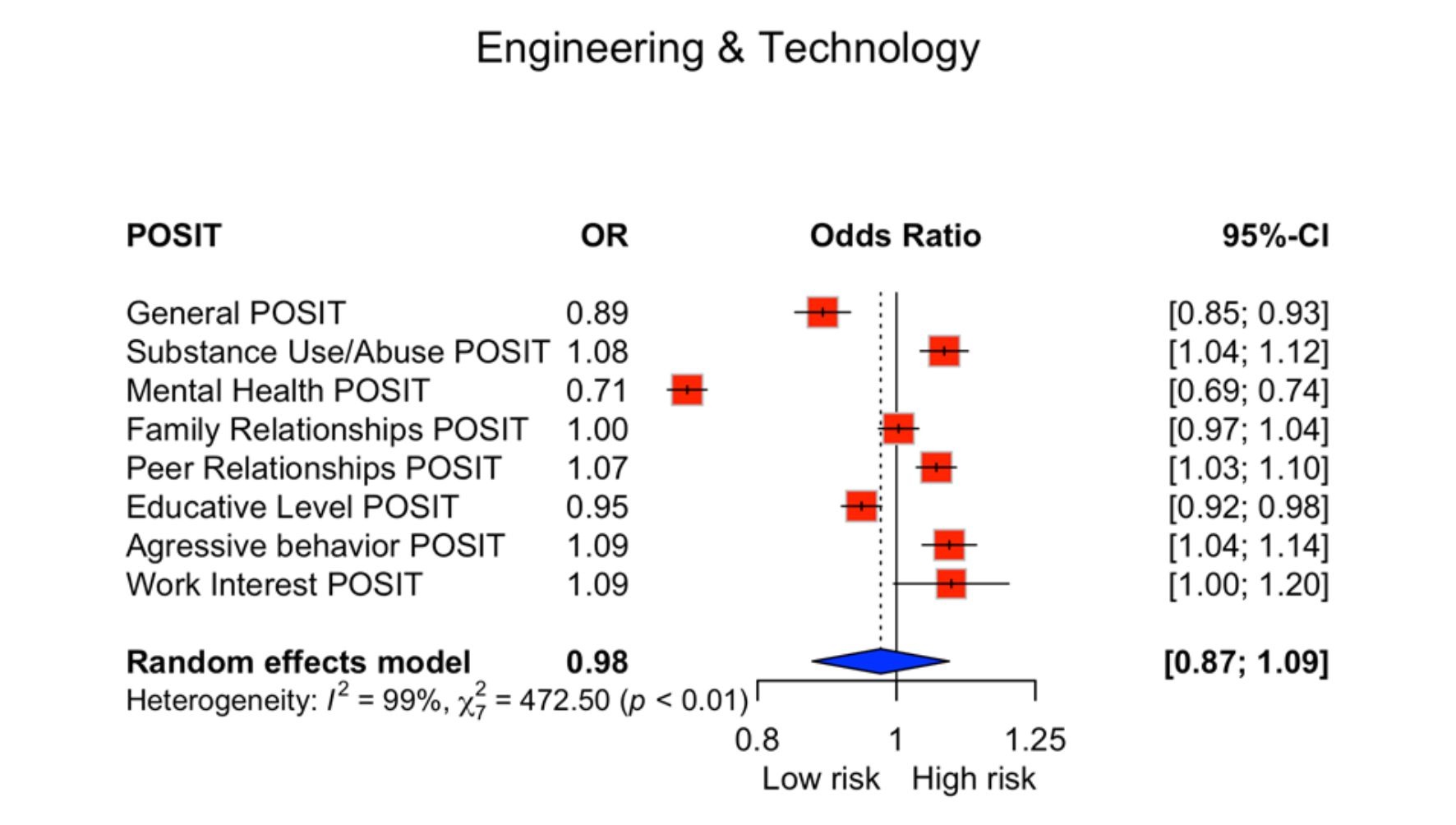

Supplement: SUPPLEMENTARY FIGURE S5 — Odds ratios for students in Engineering and Technology. Odds ratios comparing Engineering and Technology students versus all other disciplines. Models adjusted for age and sex. OR, odds ratio; CI, confidence interval; POSIT, Problem-Oriented Screening Instrument for Teenagers. [file Image_5.JPEG]

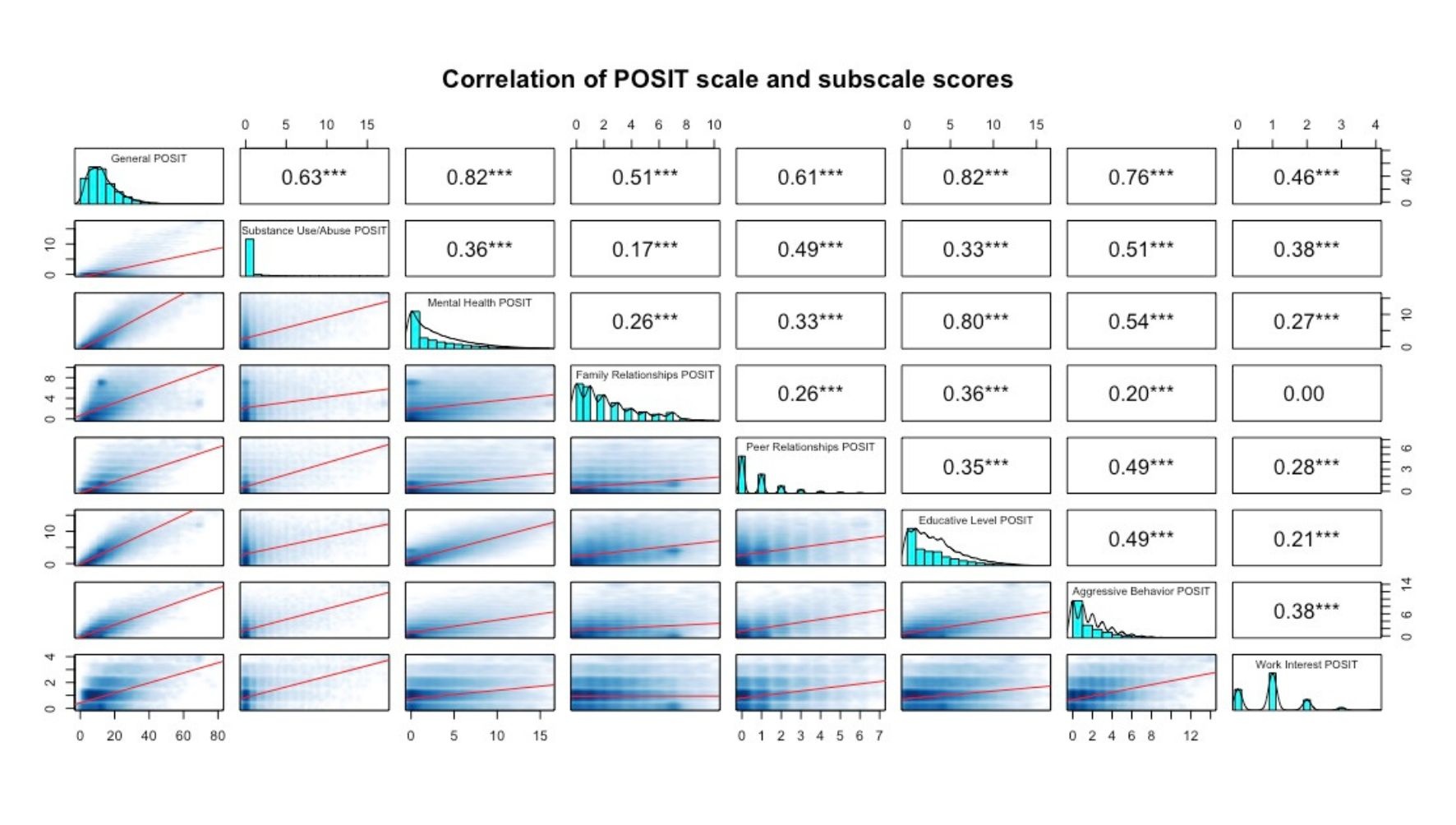

Supplement: SUPPLEMENTARY FIGURE S6 — Correlation matrix between POSIT domains. Correlation matrix showing Pearson correlation coefficients between the POSIT general score and subscales. All associations were statistically significant (p < 0.001). POSIT, Problem-Oriented Screening Instrument for Teenagers; OR, odds ratio; CI, confidence interval. [file Image_6.JPEG]
